# Requirement of Retinoic Acid Receptor β for Genipin Derivative-Induced Optic Nerve Regeneration in Adult Rat Retina

**DOI:** 10.1371/journal.pone.0071252

**Published:** 2013-08-06

**Authors:** Yoshiki Koriyama, Yusuke Takagi, Kenzo Chiba, Matsumi Yamazaki, Kayo Sugitani, Kunizo Arai, Hirokazu Suzuki, Satoru Kato

**Affiliations:** 1 Department of Molecular Neurobiology, Graduate School of Medicine, Kanazawa University, Kanazawa, Japan; 2 Department of Clinical Drug Informatics, Faculty of Pharmacy, Kanazawa University, Kanazawa, Japan; 3 Department of Biochemistry Faculty of Pharmaceutical Sciences, Hokuriku University, Kanazawa, Japan; 4 Division of Health Sciences, Graduate School of Medicine, Kanazawa University, Kanazawa, Japan; 5 Department of Medicinal Chemistry, Faculty of Pharmaceutical Sciences, Hokuriku University, Kanazawa, Japan; Dalhousie University, Canada

## Abstract

Like other CNS neurons, mature retinal ganglion cells (RGCs) are unable to regenerate their axons after nerve injury due to a diminished intrinsic regenerative capacity. One of the reasons why they lose the capacity for axon regeneration seems to be associated with a dramatic shift in RGCs’ program of gene expression by epigenetic modulation. We recently reported that (1R)-*iso*Propyloxygenipin (IPRG001), a genipin derivative, has both neuroprotective and neurite outgrowth activities in murine RGC-5 retinal precursor cells. These effects were both mediated by nitric oxide (NO)/S-nitrosylation signaling. Neuritogenic activity was mediated by S-nitrosylation of histone deacetylase-2 (HDAC2), which subsequently induced retinoic acid receptor β (RARβ) expression via chromatin remodeling *in vitro*. RARβ plays important roles of neural growth and differentiation in development. However, the role of RARβ expression during adult rat optic nerve regeneration is not clear. In the present study, we extended this hypothesis to examine optic nerve regeneration by IPRG001 in adult rat RGCs *in vivo*. We found a correlation between RARβ expression and neurite outgrowth with age in the developing rat retina. Moreover, we found that IPRG001 significantly induced RARβ expression in adult rat RGCs through the S-nitrosylation of HDAC2 processing mechanism. Concomitant with RARβ expression, adult rat RGCs displayed a regenerative capacity for optic axons *in vivo* by IPRG001 treatment. These neuritogenic effects of IPRG001 were specifically suppressed by siRNA for RARβ. Thus, the dual neuroprotective and neuritogenic actions of genipin via S-nitrosylation might offer a powerful therapeutic tool for the treatment of RGC degenerative disorders.

## Introduction

In contrast to the PNS, neurons of the adult CNS do not normally display axon regeneration after nerve injury. The neonatal nervous system of mammals retains some capacity for CNS regeneration but lose this ability after birth [1]. In newborn stages, RGCs can regrow injured axon within 1–2 weeks after birth [2], [3]. This switch of capacity for axon regeneration is associated with a dramatic shift in RGCs’ program of gene expression [4]. The normal development induces restrictive expression of growth associated genes [5] and regeneration associated genes [6]. One of cell-intrinsic change that may contribute to RGCs’ loss of regenerative potential is a decrease of histone acetylation. Histone acetylation declines in rat RGCs within 2–3 weeks after birth and decreases even further after nerve injury [7]. Thus, we hypothesize that re-expression of regeneration associated genes which show highly expression in neonatal but not in adulthood in RGCs are able to regenerate optic nerve after injury.

Genipin, an herbal iridoid, has been shown to have both neuroprotective and neuritogenic activity in PC12h cells and Neuro2a cells [8]–[12]. Recently, we extended this activity to RGC-5, retinal precursor cell line using (1R)-*iso*ropyloxygenipin (IPRG001), a long-acting genipin derivative. IPRG001 showed significant neuroprotective activity in RGC-5 cells against oxidative stress, such as hydrogen peroxide exposure [13]. Furthermore, IPRG001 sufficiently promoted staurosporine-induced neurite outgrowth from RGC-5 cells in a dose-dependent manner [14]. However, whether IPRG001 can be used to enhance axon regeneration after injury *in vivo* has not been shown. The molecular mechanism of genipin-induced neuroprotective and neurite outgrowth activity was initially believed to be neural nitric oxide synthase (nNOS)/nitric oxide (NO) -dependent because of its structural similarity to tetrahydrobiopterin, which is a cofactor for NOS enzymatic activity [15]. Indeed, both the neuroprotective and neuritogenic effects of IPRG001 in RGC-5 cells were all nNOS/NO-dependent [13], [14]. As protein S-nitrosylation is a sequential event following NO generation by nNOS activation, we focused on this modification in RGC-5 cells because of the ineffectiveness of NO/cGMP signaling [14]. However, the target proteins of S-nitrosylation for neuroprotection and neurite outgrowth were different for each activity. The neuroprotective target is Kelch-like ECH-associated protein-1 (Keap1)/NF-E2 related factor2 (Nrf2), leading to the activation of antioxidative protein expression, whereas the neuritogenic target on the RGC-5 cells is histone deacetylase 2 (HDAC-2), leading to the induction of histone acetylation and retinoic acid receptor β (RARβ) expression [13], [14]. Retinoic acid signaling plays essential roles in neural development, growth, and cellular differentiation [16] via members of the nuclear receptor family including RARs. Especially, the expression levels of RARβ are restricted in adult retina [17]. However, the role of RARβ upon adult rat optic nerve regeneration is unknown. Therefore, in the present study, we examined the neuritogenic properties of genipin on optic nerve regeneration in adult rat retinal ganglion cells (RGCs) *in vivo* after nerve injury. IPRG001 induced RARβ expression in adult rat RGCs through the NO/S-nitrosylation pathway. Concomitant with RARβ expression, IPRG001 successfully regenerated optic axons from matured rat RGCs *in vivo*. These findings may be useful for development of novel therapeutic strategies for CNS regeneration.

## Materials and Methods

### Ethics Statement

All animal care and handling procedures were approved by the Animal Care and Use Committee of Kanazawa University (No.111848). All surgery was performed under sodium pentobarbital anesthesia, and all efforts were made to minimize suffering.

### Chemicals

Genipin was purchased from Wako Pure Chemical Industries, Ltd. (Osaka, Japan). (1R)-*iso*Propyloxygenipin (IPRG001) was synthesized from genipin, as previously described [18], and dissolved in dimethylsulfoxide. 2-(4-carboxyphenyl)-4, 4, 5, 5-tetramethylimidazoline-1-oxyl-3-oxide sodium salt (c-PTIO) and S-ethyl-N-[4-(trifluoromethyl) phenyl] isothiourea (ETPI), were obtained from Alexis Co. (San Diego, CA, USA).

### Animals and Surgery

Sprague–Dawley male rats (postnatal 1 day to adult (body weight, 250–300 g)) were used throughout this study. Rats were by intraperitoneal injection of sodium pentobarbital (30–40 mg/kg body weight). Intravitreal injections of various reagents were performed with a Hamilton microsyringe (Hamilton Syringe, Whitteier, CA, USA) 30G needle. Injections were performed by the sclera and retina with a needle 1–2 mm superior to the optic nerve head to avoid lens injury which itself leads to considerable regeneration as a consequence of one or more trophic factors associated with inflammatory cells [19]. As the control of each experiment, we injected vehicles to eye ball. The volume of injection was set at 5 µl of total volume after pre-suction of the same volume of vitreal fluid. After lateral canthotomy of rats, the conjunctiva was incised laterally to the cornea, the retractor bulbi muscle was separated, and the optic nerve exposed under the binocular-operating microscope. Optic nerve was crushed 2 mm behind the eye with angled jeweler’s forceps (Dumont # 5) for 10 sec, avoiding injury to the ophthalmic artery as previously described [20]. Rats were housed in clear plastic cages and maintained under the 12 h light/dark cycles at 23°C.

### Retinal Explant Culture

Rat retinal explant cultures were performed as previously described [14]. Retinas were cut into small pieces (0.5 mm squares) and cultured in medium containing DMEM (Sigma-Aldrich, St Louis, MO, USA), 10% fetal bovine serum and penicillin–streptomycin (100 µg/ml–100 U/ml, respectively) in collagen gel (Cellmatrix; Nitta Gelatin, Osaka, Japan) on a 35 mm culture dish. We observed neurite outgrowth in each explant from total 30–40 explants per dish using phase contrast microscopy. Positive neurite outgrowth was defined on the basis of the length (>200 µm) of the neurites, following the description in our previous study [14]. We showed the percentage of explants with positive neurites. Four independent experiments were repeated.

### Immunohistochemistry

Tissue fixation and cryosectioning were performed as previously described [21]. Briefly, the eyes and optic nerves were isolated and fixed in 4% paraformaldehyde containing 0.1 M phosphate buffer (pH 7.4) and 5% sucrose for 2 h at 4°C. Sucrose concentration was gradually increased from 5 to 20%. The eyes were then embedded in optimal cutting temperature compound, Tissue Tek (Sakura Finetek, Tokyo Japan) and cryosectioned at 12 µm thickness. The frozen sections were mounted and blocked with Blocking One (Nacalai Tesque, Japan) and retinal sections were incubated with primary anti-nNOS (1∶500), anti-RARβ (1∶500) (Santa Cruz Biotechnology, Santa Cruz, CA, USA), anti-NeuN (1∶500, Millipore, Billerica, MA, USA) and anti-acetyl histone H3 (1∶500, Cell Signaling Technology, Tokyo, Japan) antibodies. The sections were then incubated with Alexafluoro anti-IgG (Molecular probe, Eugene, OR, USA).

### NADPH Diaphorase Staining

Tissue fixation and cryosectioning were performed as previously described [22]. The frozen sections were mounted onto silane-coated glass slides and air-dried. The slides were then brought to 23°C and incubated overnight in 0.1 M Tris-HCl (pH 8.0) containing 0.3% Triton-X 100. Each sample was stained in buffer containing NADPH and 4-nitroblue tetrazolium chloride (Roche Diagnostics Corporation, Indianapolis, IN, USA) for 2–3 h at 37°C.

### Subcellular Fractions for the Extraction of RARβ Protein

Retinas were lysed in hypotonic buffer containing 10 mM HEPES-KOH (pH7.9), 10 mM KCl, 1.5 mM MgCl_2_, 1 mM DTT, 0.5 mM phenylmethylsulfonyl fluoride, protease inhibitor cocktail and centrifuged at 10,000 g for 15 min at 4°C. The supernatants were used as the cytoplasmic fraction and the pellets were incubated with a nuclear lysis buffer containing 20 mM HEPES-KOH (pH7.9), 400 mM NaCl, 1.5 mM MgCl_2_, 0.2 mM EDTA, 1 mM DTT, 5% glycerol, and protease inhibitor cocktail (Sigma-Aldrich) for 30 min on ice. The lysates were centrifuged at 18,000 g for 15 min at 4°C. The supernatants contained the nuclear fraction. Immunoblotting analysis of β-actin and histone H4 was performed to ensure no contamination of cytoplasmic and nuclear fractions.

### Western Blot Analysis

Retinal explants or retinas from various conditions were extracted and 30 µg of protein were subjected to polyacrylamide gel electrophoresis using a 5–20% gradient gel as previously described [22]. The separated proteins were transferred to a nitrocellulose membrane and incubated with primary and secondary antibodies (Santa Cruz Biotechnology). Protein bands (nNOS (1∶500), RARβ (1∶500)) were detected using a BCIP/NBT Kit (Funakoshi, Tokyo, Japan). Antibodies against β-actin (1∶500, Gene Tex, San Antonio, TX) and histone H4 (1∶500, Cell Signaling Technology, Tokyo, Japan) were used as an internal standard. Protein bands isolated from cells cultured under various conditions were analyzed densitometrically using Scion Image Software (Scion Corp. Frederick, MD, USA). All experiments were repeated at least three times.

### S-Nitrosylation Analyses of HDAC2

S-Nitrosylation of HDAC2 was assessed by a modified of biotin switch assay [23] using the S-nitrosylated Protein Detection Assay Kit (Cayman Chemical, Ann Arbor, MI). Retinas exposed to agents for 1 day were harvested and lysed at 4°C. Free thiols were blocked by adding S-methyl methanethiosulfonate and biotinylation of nitrosothiols was performed using maleimide-biotin. Biotinylated proteins were further purified by overnight incubation with neutravidin-coupled agarose beads (Pierce-Thermo Scientific, Rockford, IL, USA). After incubation, beads were washed three times with PBS. Isolated proteins were recovered from beads by the addition of Laemmli sample buffer, and heated at 85°C for 10 min. The amount of S-nitrosylated HDAC2 protein in the samples was analyzed by western blot analysis using anti-HDAC2 antibody (1∶500, Cell Signaling Technology, Tokyo, Japan).

### Application of siRNA for RARβ

Small interfering RNA (siRNA) for the target region of RARβ mRNA were as follows: 5′-GGAGCCGACUGCAAAUACAAG-3′ (sense); 5′-UGUAUUUGCAGUCGGCUCCAA-3′ (antisense) (Sigma-Aldrich, Japan); and a randomly shuffled sequence: 5′-AGUCGUCGUAUACGGUAUAUC -3′ (sense); 5′-UAUACCGUAUACGACGACUUC -3′ (antisense). In retinal explant culture, transfection of siRNA (100 pmol) into retinal explants was carried out using Lipofectamine 2000 (Invitrogen Corporation, Carlsbad, CA, USA) for 4 h before culture. In rat retina, knockdown of RARβ expression was performed by injecting siRNA (2 µg) using a 30-gauge syringe to eyes as described [24], [25]. After injection, five square 80 V pulses of 50 ms duration with 950 ms intervals were applied by NEPA21 (Nepagene, Chiba, Japan) with forcep-type electrodes. After 5 days of electroporation, retinal cells were cultured, and optic nerves were crushed.

### Quantitation of Optic Nerve Regeneration *in vivo*

Rats were sacrificed at 14 days after optic nerve injury and were perfused with 4% paraformaldehyde. Optic nerves were impregnated with 5% and then 30% sucrose, embedded in Optimal Cutting Temperature compound (Sakura Finetechnical, Tokyo, Japan) and cut into longitudinal sections of 14 µm thickness. Regenerating optic axons were visualized by staining with mouse anti-GAP43 antibody (1∶250, Santa Cruz Biotechnology, Santa Cruz, CA) followed by a fluorescently-labeled secondary antibody and captured by fluorescent microscopy (BZ-9000, Keyence, Osaka, Japan). Axons were counted manually in at least 8 sections per conditions (6 rats of each treatment) at prespecified distances (250 µm and 500 µm) away from the injury site. The numbers of regenerating axons were counted as described by Leon et al. [19].

### Counting of Surviving RGCs

RGC survival was evaluated in flat-mounted retinas by immunohistochemistry using a mouse antibody against βIII-tubulin (TUJ1, R&D Systems, Inc., Minneapolis, USA 1∶500), followed by a fluorescent secondary antibody after 10 days of injury. Images of 8 prespecified retinal areas of 3 mm away from the optic disc (middle region of the retina) were captured by fluorescent microscopy (under x200 magnification; E600, Nikon) and positive cells were counted using ImageJ software (Wayne Rasband, NIH, Bethesda, MD). Cell densities were calculated as mean values of surviving RGCs per 0.14 mm^2^ in the 8 specified areas. Data are presented as means ± S.E.M on five rats per group.

### Statistics

All results were reported as mean ± SEM for 3–5 experiments. Differences between groups were analyzed using one-way ANOVA, followed by Dunnett’s multi-comparison test with PASW Software (SPSS Inc., Chicago, IL, USA). P values <0.05 were considered statistically significant.

## Result

### Correlation between Axonal Regeneration and RARβ Expression in the Developing Rat Retina

**Figure 1:**
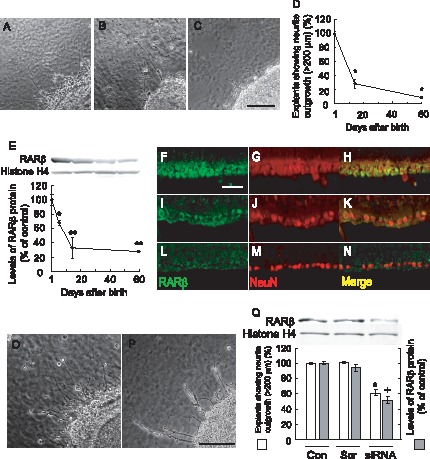
Correlation between axon outgrowth and RARβ expression in rat retina after birth. (A) P1 retinal explant, (B) P14 retinal explant, (C) P60 retinal explant, Scale = 100 µm. (D) Quantification of axon outgrowth from rat retinal explants at various days after birth. Y axis shows % of explants with neurites longer than 200 µm. *P<0.01 vs P1 retina. (n = 3, 5 rats of each stage). (E) Quantification of RARβ expression in rat retina during development. *P<0.05, **P<0.01 vs P1 (n = 3). (F–N) RARβ and NeuN expression in the rat retina during development. (F, I, L) RARβ expression at P1 (F), P14 (I) and P60 (L). Scale = 50 µm. (G, J, M) NeuN-positive RGCs at P1 (G), P14 (J), and P60 (M). (H, K, N) Merged images of each day at P1 (H), P14 (K), and P60 (N). (O) P1 retina treated with scrambled siRNA (P) siRNA for RARβ in P1. Scale = 100 µm. (Q) siRNA of RARβ suppressed expression of RARβ and neurite outgrowth in P1 retina. *P<0.01 vs vehicle control (Con) and scrambled siRNA(Scr) (RARβ expression), ^+^P<0.01 vs Con and Scr (neurite outgrowth), (n = 3, 5 rats of each treatment).

Newborn rat retina at postnatal day 1 (P1) showed spontaneous axonal regeneration in explant cultures forming long neurites more than 200 µm in length (Fig. 1A). In contrast, rat retina at postnatal day 14 (P14, Fig. 1B), and day 60 (P60, Fig. 1C) lost the capacity for axonal regeneration in explant culture. Figure 1D shows a graphical representation of neurite outgrowth in P1, P14 and P60 rat retinal explant cultures. In P14 rat retina, neurite outgrowth was about 30% of P1 retina. In P60 rat retina, neurite outgrowth was less than 10% of P1 retina. To ascertain the levels of retinoic acid receptor β (RARβ) in rat retina after birth, we performed western blot analysis in the total retina at P1, P5, P14 and P60. Figure 1E shows a graphical representation of RARβ protein expression in developing rat retinas. In P5 rat retina, the levels of RARβ expression were 68% of P1 rat. In P14 rat retina, the level of RARβ expression was about 30% of P1 rat. In P60 rat retina, the expression was less than 30% of P1 rat. Histone H4 protein did not change during this period (Fig. 1E). Similarly, we performed immunohistochemistry for RARβ protein expression in the developing rat retina. At P1 rat retina, strong immunoreactivity for RARβ could be seen in the retinal ganglion cell layer (Fig. 1F). The localization of RARβ in RGCs was further confirmed using anti-NeuN, a potent marker of RGCs [26], [27] (Fig. 1G and Fig. 1H merged). In P14 rat retina, RARβ expression in the RGCs was decreased compared to P1 rat retina (Figs. 1 I, J, K). In adult P60 rat retina, RARβ expression in the RGCs was barely visible (Figs. 1 L, M, N). From these results similarity between neurite outgrowth and RARβ expression in development retina, we next examined the effect of RARβ knockdown with retinal explant culture on neurite outgrowth. A specific siRNA for RARβ suppressed the neurite outgrowth to 63% (RARβ expression to 52%) compared with no treated control of P1 retina (Figs. 1P and Q), whereas scrambled RNA did not suppress both neurite outgrowth and RARβ expression (Fig. 1O and Q).

### Increased NOS Activity and nNOS Expression in rat RGCs by IPRG001 Treatment

**Figure 2:**
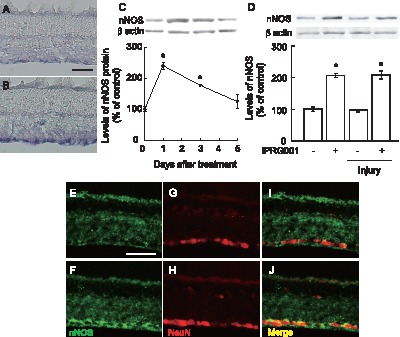
IPRG001 activates NADPHd staining and nNOS protein expression in the rat retina. (A, B) NADPHd staining in the retina increased at 1 day post-treatment with 100 pmol/eye of IPRG001 (B) as compared to vehicle control (A). Scale = 100 µm. (C) Western blot analysis of nNOS protein after treatment of IPRG001. *P<0.01 vs control (n = 3). (D) Levels of nNOS expression by IPRG001 with or without optic nerve injury. *P<0.01 vs control (n = 3). (E–J) nNOS and NeuN expression in the retina after treatment of IPRG001. (E and F) Immunoreactivity of nNOS protein was increased in RGCs at 1 day after IPRG001 treatment (F) compared to vehicle control (E). Scale = 50 µm. (G and H) NeuN staining of E and F. (I and J) Merged images.

As we showed that IPRG001 induced neurite outgrowth in RGC-5 cells through nNOS/NO signaling [14], we examined NOS activity and nNOS expression in the rat retina after intraocular injection of IPRG001. To examine NOS activity, we studied histochemical staining of NADPH diaphorase activity, which was associated with NOS activity [22], [28]. Intraocular injection of IPRG001 (100 pmol/eye) significantly increased NADPH diaphorase staining in RGCs (Fig. 2B) 1 day after treatment compared to vehicle control retina (Fig. 2A). Next, we studied the effects of IPRG001 on nNOS protein expression in the adult rat retina by western blot. Intraocular IPRG001 significantly increased nNOS protein levels 2.5-fold at 1 day after treatment compared to vehicle control (Fig. 2C). The increase in nNOS expression returned to control levels by 5 days after treatment. Levels of β-actin expression did not change during this period (Fig. 2C). We further compared nNOS expression with or without IPRG001 injection at 1 day following optic nerve injury (Fig. 2D). Optic nerve injury did not change the levels of nNOS expression at this period compared to control as reported by Lee et al. [29]. IPRG001 increased a similar level of nNOS protein at 1 day after treatment with or without optic nerve injury. To determine nNOS protein expression by IPRG001, we performed double-staining in adult rat retina with anti-nNOS and anti-NeuN antibodies. At 1 day after IPRG001 treatment, a strong immunoreactivity for nNOS could be seen in RGCs (Figs. 2F, H, J) compared to the control retina (Figs. 2E, G, I). The localization of increased expression of nNOS by IPRG001 was also observed in RGCs after nerve injury (data not shown).

### Increased RARβ Expression in Adult Rat RGCs with IPRG001 Treatment through nNOS/NO Signaling

**Figure 3:**
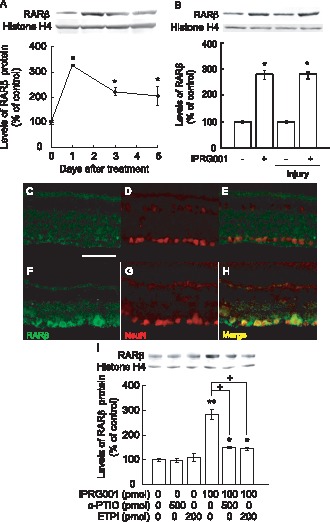
Upregulation of RARβ expression in RGCs after IPRG001 treatment. (A) Western blot analysis of RARβ protein expression increased at 1 day after treatment of IPRG001. *P<0.01 vs control (n = 3). (B) Levels of RARβ expression by IPRG001 with or without optic nerve injury. *P<0.01 vs control (n = 3). (C–H) RARβ and NeuN expression in the retina after treatment of IPRG001. (C and F) Immunoreactivity of RARβ protein was increased in RGCs 1 day after treatment with IPRG001 (F) compared to vehicle control (C). Scale = 50 µm. (D and G) NeuN staining of C and F. (E and H) Merged images. (I) nNOS/NO-dependent expression of RARβ by IPRG001. *P<0.05, **P<0.01 vs control, ^+^P<0.01 vs IPRG001 treatment alone (n = 3).

In our previous study, we found that IPRG001 induced RARβ expression through nNOS/NO signaling in RGC-5 cells [14]. In this study, we investigated the effects of IPRG001 on RARβ expression in the adult rat retina. The changes of RARβ protein expression in the rat retina were examined for 5 days after treatment (Fig. 3A). RARβ rapidly increased by 3-fold of control levels in the retina at 1 day and gradually declined to lower levels by day 5 post-treatment (Fig. 3A). Histone H4 expression did not change during this period. IPRG001 induced a similar level of RARβ protein at 1 day after treatment with or without optic nerve injury (Fig. 3B). Next, we performed immunohistochemistry for RARβ protein expression in the rat retina after treatment of IPRG001. In vehicle control retina, weak RARβ expression was seen in RGCs with anti-RARβ and anti-NeuN antibodies (Figs. 3C, D, E). Intraocular IPRG001 (100 pmol/eye) increased RARβ expression in RGCs at 1 day post-treatment (Figs. 3F, G, H). The localization of RARβ expression by IPRG001 was seen in RGCs at 1 day after nerve injury (data not shown). The induction of RARβ expression by IPRG001 in the rat retina was significantly blocked by a NO scavenger, c-PTIO (500 pmol) or a specific nNOS inhibitor, ETPI (200 pmol) (Fig. 3I). Neither c-PTIO nor ETPI had any effects on RARβ expression in vehicle control retina. These data strongly indicate that the IPRG001-induced RARβ expression in the adult rat RGCs was nNOS/NO-dependent.

### IPRG001-induced S-nitrosylation of Histone Deacetylase 2 (HDAC2) and Acetylation of Histone H3

**Figure 4:**
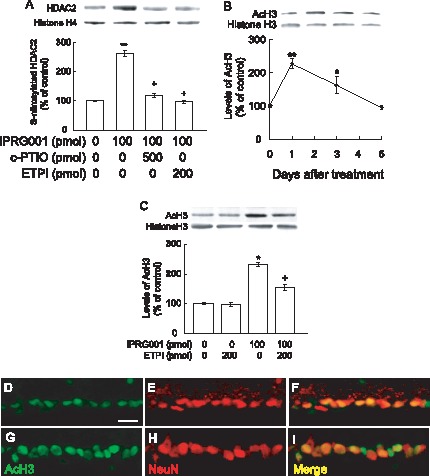
S-Nitrosylation of HDAC2 and the acetylation of histone H3 in the rat retina by IPRG001. (A) S-Nitrosylation of HDAC2 1 day after intraocular administration of IPRG001. NO scavenger, c-PTIO or nNOS inhibitor, ETPI was treated 1 h before IPRG001 treatment. Biotinylated proteins were mixed with avidin beads, eluted and analyzed by western blotting with anti-HDAC2 antibody. **P<0.01 vs control. ^+^P<0.01 vs IPRG001 treatment alone (n = 3). (B) Levels of acetylated histone H3 (AcH3) after treatment of IPRG001. *P<0.05, **P<0.01 vs control (n = 3). (C) nNOS dependent histone H3 acetylation by IPRG001. ETPI was treated 1 h before IPRG001. *P<0.01 vs control. ^+^P<0.01 vs IPRG001 treatment alone (n = 3). (D–I) Immunohistochemistry of AcH3 and NeuN in rat RGCs. Levels of AcH3 were increased 1 day after IPRG001 treatment (G–I) compared to vehicle control (D–F). (D and G) Immunoreactivity of AcH3. (E and H) NeuN staining. (F and I) Merged images. Scale = 20 µm.

In our previous study, we showed that IPRG001-induced nNOS/NO signaling occurred after the S-nitrosylation of HDAC2 leading to increased RARβ expression and neuritogenesis in RGC-5 cells [14]. Therefore, we investigated the S-nitrosylation of HDAC2 after IPRG001 treatment. Figure 4A shows a 2.6-fold increase in the S-nitrosylation of HDAC2 at 1 day after IPRG001 (100 pmol/eye) treatment. The S-nitrosylation of HDAC2 was also nNOS/NO-dependent. c-PTIO or ETPI significantly blocked HDAC2 S-nitrosylation (Fig. 4A). Figure 4B illustrates a graphical representation of acetylated histone H3 (AcH3) with IPRG001 (100 pmol/eye) treatment. IPRG001 increased AcH3 2.3-fold in the rat retina at 1 day and returned to control levels by 5 days post treatment. The increase of AcH3 by intraocular IPRG001 was almost blocked by ETPI (Fig. 4C). Immunohistochemical staining revealed that IPRG001 significantly enhanced immunoreactivity of AcH3 in rat RGCs at 1 day after treatment (Figs. 4 G, H, I) compared to vehicle control retina (Figs. 4D, E, F). The data indicate that there is a good correlation between the S-nitrosylation of HDAC2 and AcH3 in the rat retina for 1day treatment of IPRG001.

### Intraocular IPRG001 Promotes Optic Nerve Regeneration from Adult Rat RGCs through a RARβ-dependent Mechanism after Nerve Injury

**Figure 5:**
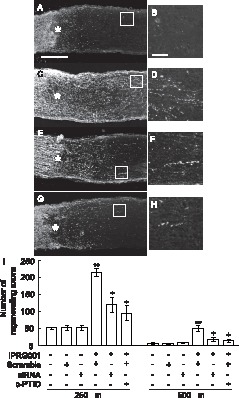
IPRG001-induced rat optic nerve regeneration under RARβ-dependent conditions *in vivo*. (A, C, E, G) Longitudinal sections of the adult rat optic nerve showing GAP-43 positive axons extending over the injury site (asterisks) after 2 weeks after optic nerve injury. (B, D, F, H) Enlarged image of the area enclosed within the white box of A, C, E, G. Scale = 50 µm. (A) Vehicle control, Scale = 250 µm. (C) IPRG001, (E) IPRG001 plus siRNA for RARβ. (G) IPRG001 plus c-PTIO (500 pmol). (I) Quantification of axonal regrowth at two indicated proximal points from the injury site (250 µm and 500 µm). **P<0.01 vs control, ^+^P<0.01 vs injury plus IPRG001 (n = 8, 6 rats per each group).

As IPRG001 enhanced neurite outgrowth from retinal explants *in vitro* [14], we further confirmed this activity *in vivo* preparation after nerve injury. Intraocular IPRG001 (100 pmol/eye) induced optic nerve regeneration *in vivo* (Fig. 5C) compared to vehicle control (Fig. 5A), which was revealed by GAP43 staining. siRNA for RARβ significantly suppressed the effects of IPRG001 (Fig. 5E). c-PTIO also attenuated the IPRG001-induced optic nerve regeneration (Fig. 5G) although c-PTIO alone did not change the optic nerve regeneration of no treatment (data not shown). Figures 5B, D, F, H show enlarged images of the areas enclosed within the white boxes in Figs. 5A, C, E, G, respectively. IPRG001 (Fig. 5D) showed many regenerating fibers compared to control (Fig. 5B), IPRG001 plus siRNA (Fig. 5F), or IPRG001 plus c-PTIO (Fig. 5H). Figure 5I illustrates the quantitative data of optic nerve regeneration *in vivo* at 250 µm and 500 µm away from the crush site of optic nerve (asterisk). At both sites, siRNA for RARβ significantly cancelled the effects of IPRG001. In contrast, scramble siRNA did not affect the axon elongation effects of IPRG001. siRNA and scramble siRNA alone did not affect the basal level of optic nerve regeneration without IPRG001 treatment (Fig. 5I).

### RARβ Induction by IPRG001 did not Promote RGCs Survival after Nerve Injury

**Figure 6:**
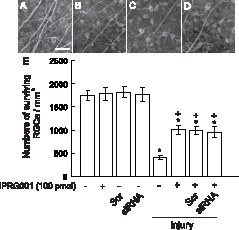
No effect of RARβ expression on RGCs survival induced by IPRG001. (A-D) Surviving RGCs stained by TUJ1 in whole mount retina. (A) Vehicle control. Scale = 50 µm. (B) 10 days after injury. (C) Injury plus IPRG001. (D) Injury plus IPRG001 with RARβ-specific siRNA. (E) Quantification of surviving RGCs after nerve injury with or without siRNA for RARβ. *P<0.01 vs control, ^+^P<0.01 vs injury (n = 8).

IPRG001 sufficiently rescued rat RGCs against optic nerve injury through antioxidative mechanism [13]. Here, we further examined whether or not the protective effect of IPRG001 was mediated through a RARβ expression mechanism. We therefore counted the number of surviving RGCs by staining of anti-TUJI antibody after nerve injury. Optic nerve injury drastically reduced the number of surviving RGCs at 10 days after nerve injury (Fig. 6B) as compared to the vehicle control (Fig. 6A). IPRG001 certainly rescued the RGCs cell death after injury (Fig. 6C). However, neither siRNA for RARβ (Fig. 6D) nor scrambled siRNA affects this protection of RGCs cell death by IPRG001 (Fig. 6E). Fig. 6E illustrates a graphical representation of the number of surviving RGCs after optic nerve injury with or without siRNA for RARβ. These results suggest that this RARβ induction by IPRG001 is targeted only to the neuritogenic action, not to survival action.

## Discussion

### A Correlation between Neurite Outgrowth and RARβ Expression in the Developing Rat Retina

Immature retina has a higher capacity for optic nerve regeneration rather than mature retina *in vitro* [30]. However, the rat retina gradually loses this capacity for regeneration with development, although the reason remains unsolved. Goldberg et al. [3] reported that rat retinal ganglion cells (RGCs) of embryonic day 20 (E20) had the most intensive capacity for extending optic axons and they showed a higher extension of optic axons 7-fold than the RGCs at postnatal day 8 (P8). We confirmed these results with developing rat retinas of postnatal days 1, 14 and 60 (Fig. 1). It is also known that retinoic acid (RA) signaling is involved in neural development and regeneration regulated through nuclear RA receptors (RARs). In the dorsal root ganglia and spinal cord, a particular role of RARβ expression was implicated in nerve regeneration after nerve injury. Grondona et al. [31] reported that RARβ expression in the mouse retina could be seen in RGCs from postnatal days 0 to 21 (P0–P21). Mori et al. [32] also reported RARβ expression in the developing rat retina. RARβ expression in P14 or P60 rat RGCs was less than 30% of those at P1 (Fig. 1E). The cellular localization of RARβ in RGCs was confirmed by double-staining of NeuN labeling [26]. This time course of RARβ expression in the developing rat retina was very comparable to that of our data (cf. Fig. 1D and Fig. 1E). Furthermore, we indicated that specific siRNA for RARβ significantly suppressed neurite outgrowth in P1 retina. These results suggest that RARβ is one of the important molecules for neuritogenesis in developing retina. The data of developing rat retina further encouraged us to investigate the neuritogenic activity of RARβ in adult rat retina *in vivo*.

### RARβ Expression Induced by IPRG001 is Mediated via NO/S-nitrosylation of HDAC2

In the previous study with RGC-5 cells, IPRG001 promoted staurosporine-induced neurite outgrowth in an nNOS/NO-dependent manner [14]. RGC-5 cells were treated with staurosporine to arrest cell growth and induce cell differentiation [33]. RGC-5 cells are known as neuronal precursor cells of retinal origin. Recently, Van Bergen et al. [34] reported the re-characterization of RGC-5 cells. Differentiated by staurosporine, RGC-5 cells express the RGCs markers, Thy1 and the NMDA receptor [35], [36]. Thus, we used staurosporine as a reagent for RGC-5 differentiation as an *in vitro* model of RGCs. However, whether IPRG001 can be used to enhance axon regeneration of RGCs after injury *in vivo* has not been shown. The neurite outgrowth activity of IPRG001 in RGC-5 cells is mediated by the S-nitrosylation of HDAC2. It is well known that the inhibition of HDAC2 induces chromatin remodeling that leads to the transcriptional activation of genes including RARβ [37]–[39]. Nott et al. [40] further reported that the S-nitrosylation of HDAC2 also caused chromatin remodeling in the neural cells like HDAC2 inhibition. In our examination of RGC-5 cells *in vitro*, the induction of RARβ by IPRG001 was nNOS/NO- and HDAC2 S-nitrosylation-dependent [14]. In the present study, IPRG001 significantly S-nitrosylated HDAC2 in rat RGCs at 1 day post treatment. The HDAC2 S-nitrosylation in the rat retina was also nNOS/NO-dependent. A NO scavenger and nNOS inhibitor clearly suppressed RARβ expression. HDAC inhibitor, which can induce histone H3 acetylation (AcH3) and RARβ expression [38], dramatically promotes neurite outgrowth in retinal explant culture [41]. Moreover, it has reported that HDAC inhibitor, trichostatin A strongly induced histone H3 acetylation, neuritogenesis in RGCs [7], [42] and RARβ expression [43], [44]. Our data of IPRG001 well agree with their results. IPRG001 increased AcH3 in the rat RGC 2.6-fold at 1 day after optic nerve injury by western blot analysis (Fig. 4C). This increase of AcH3 was nNOS-dependent. ETPI, a nNOS inhibitor completely blocked the IPRG001-induced increase of AcH3. These data strongly indicate that IPRG001 leads chromatin remodeling like an HDAC2 inhibitor through an NO/S-nitrosylation mechanism. Recently, Gaub et al. [7] reported that the levels of histone acetyltransferase P300 protein and histone acetylation (AcH3) in the rat RGCs decreased with maturation. This property might be one of the reasons why mammalian optic nerves lost the capacity for optic nerve regeneration after nerve injury [41].

### Role of RARβ Expression in Rat RGCs for Optic Nerve Regeneration

In general, successful CNS regeneration after nerve injury is mainly dependent on neuritogenesis. As for neuronal cell survival, the overexpression of Bcl-2, an antiapoptotic molecule in mouse RGCs, sufficiently maintains the cell survival of RGCs, but does not promote axonal regeneration from RGCs [45], [46]. There are many reports that RARβ expression is involved in axonal regeneration in the CNS neurons, including the spinal cord, dorsal root ganglia [47]–[49] and we could show the important role of RARβ expression during optic nerve regeneration in this study. On the other hand, the lack of regenerative capacity is attributable in part to inhibitory factors in myelin [50]. There are a few inhibitory environments on neonatal axonal regeneration within 2 days after birth [51]. Myelin basic protein, a marker protein of myelin, start to express from few days after birth and inhibitory factors from myelin inhibit axon regeneration [52]. Interestingly, retinoic acid/its receptor, RARβ counteracts myelin-dependent inhibition of axon outgrowth by repression of Lingo-1, one of the coreceptor of inhibitory Nogo receptor [53]. Although we do not know the exact mechanism behind AcH3-induced RARβ expression and its downstream signaling pathway, the cooperation with histone acetyltransferase and RAR is involved in promotion of gene expression and cell differentiation in the spinal motor neuron development [54], [55]. Further studies are needed to elucidate the mechanism of axon outgrowth through AcH3/RARβ expression. On the other hand, there are very few reports of RARβ expression resulting in neuroprotection or survival in neurons [56]. To know the effects of RARβ induction on IPRG001-induced RGCs survival after nerve injury, we used specific siRNA for RARβ. In our experimental conditions, siRNA for RARβ did not affect the neuroprotective effects of IPRG001 in rat RGCs after injury (Fig. 6). These results suggested that cell survival effect by IPRG001 in RGCs after injury was irrelevant to RARβ expression by IPRG001. In our previous study [13], IPRG001 supports RGC survival *in vivo* after optic nerve injury through the S-nitrosylation of another target protein, Keap1, leading to the induction of an antioxidative protein, heme oxygenase-1. Thus RARβ induction by IPRG001 seems to be unconnected with the survival effect against RGCs after injury. The present results in connection with our previous study [14] strongly indicate that IPRG001 activates nNOS/NO/S-nitrosylation signaling, which leads to distinct neuroprotective and neuritogenic actions on damaged adult rat RGCs *in vivo*. The concurrent neuroprotective and neuritogenic actions of IPRG001 could provide a new therapeutic tool for CNS disorders, including brain ischemia, age-related brain disorders, spinal cord injury, and RGC degenerative diseases, such as glaucoma.
